# Selecting appropriate focal species for assessing the risk to birds from newly drilled pesticide‐treated winter cereal fields in France

**DOI:** 10.1002/ieam.4112

**Published:** 2019-02-11

**Authors:** Emmanuelle Bonneris, Zhenglei Gao, Amanda Prosser, Ralf Barfknecht

**Affiliations:** ^1^ Bayer AG CropScience Division Monheim‐am‐Rhein Germany; ^2^ Tier 3 Solutions GmbH Leverkusen Germany

**Keywords:** Focal species, Birds, Pesticide risk assessment, Winter cereals, Field monitoring

## Abstract

Identifying focal bird species appropriate to the situation in which a plant protection product is used is important for refined risk assessment (EFSA 2009). We analyzed the results of extensive field observations of newly drilled cereal fields in France in autumn over 2 seasons to determine real bird focal species. In 2011, birds were observed before and after drilling on wheat and barley fields drilled with imidacloprid‐treated seeds (i.e., “treatment” fields) or seeds treated with compounds other than imidacloprid (i.e., “alternative treatment” fields). Bird abundance, species richness, and diversity were significantly higher in wheat fields than barley fields; these findings led us to monitor only wheat fields in 2012. Statistical analyses did not show a significant effect of the drilling itself or between the treatment fields and the alternative treatment fields on the number and type of bird species. These results led to the pooling of 2011 data on all fields for focal species determination. Similarly, all bird monitoring data generated in 2012 before and after drilling were pooled and analyzed. Rules for determination of candidate focal species detailed in the EFSA (2009) guidance were followed. Carrion crow, wood pigeon, gray partridge, skylark, common starling, and pied wagtail were the bird species most frequently observed on wheat fields. This list of candidate species was processed to determine the most relevant focal species according to the method of Dietzen et al. (2014); this process resulted in the selection of skylark, gray partridge, wood pigeon, and pied wagtail as focal species to assess risks to birds for pesticides applied during drilling of winter cereals in France (September through November). Integr Environ Assess Manag 2019;00:000–000. © 2018 The Authors. *Integrated Environmental Assessment and Management* published by Wiley Periodicals, Inc. on behalf of Society of Environmental Toxicology & Chemistry (SETAC)

## Key Points

- We identified real bird focal species important in higher‐tier risk assessments for plant protection products applied during drilling of winter cereals in France (September–December) such as the skylark, the gray partridge, the wood pigeon, and the pied wagtail.
- We used a large data set of bird observations performed in 126 newly drilled cereal fields in autumn in France over 2 years.
- We focused our data analysis on immediate postdrilling period important to identify relevant bird focal species.
- We followed rules detailed in the Guidance Document for Bird and Mammals (2009) for determination of candidate bird focal species and process the list of candidate species according to the method of Dietzen et al. (2014) to determine the most relevant real focal species.

## INTRODUCTION

Plant protection products in Europe are regulated to balance their benefits with the possibility of damage to the environment (Regulation EC 1107/2009; EU 2009). In order for products to be registered for use, the risk to nontarget animals must be assessed; for birds and mammals, this assessment is done following European Food Safety Agency (EFSA) guidance (EFSA 2009). If an initial screening assessment step indicates that there may be a risk to nontarget organisms, further “higher‐tier” steps are carried out to further refine the character of the risk. One possible refinement mentioned in EFSA (2009) is the use of field effect studies, which refers both to studies of effects following experimental pesticide applications (i.e., applications made as part of a regulatory study) and also to “active monitoring” of effects following applications of authorized products in agricultural practice. “Active monitoring studies” are carried out postregistration and hence assess the potential effects of the plant protection product under normal commercial use. Additional information may also come from reactive schemes in which members of the public, farmers, and growers report incidents that may (or may not) be linked to the use of a particular pesticide (Berny 2007; Millot et al. 2017). Aims of the postauthorization monitoring studies include obtaining data for refinement steps in a risk assessment to support “weight of evidence” evaluations and to confirm the safety of the registered product. Postregistration monitoring studies can be extensive in comparison to studies performed before authorization of the product, which can only be conducted on a small number of experimental fields. From the postregistration monitoring, data obtained on a larger number of sites can be used in a consistent way to assist in the regulatory decision‐making process. Additionally, bird survey data sets could be used to determine, in general, the relevant bird focal species in a crop at the period of use of the product (CSL 2009; MNHN 2012; Dietzen et al. 2014). Such data could then help to identify the species to be followed during postregistration monitorings and provide guidance for targeted stewardship activities.

Focal species, as defined by EFSA (2009), are real species that occur regularly in a particular crop and are protective (i.e., are representative and cover the risk) of other species that might be exposed to pesticide applications in the field. EFSA (2009) gives generic focal species for consideration in lower‐tier risk assessments but does not propose real focal bird species for refinement steps. Hence it can be important to establish relevant focal species in risk assessment, introducing greater realism for refined risk assessments, when needed. Various authors (e.g., Crocker and Irving 1999; CSL 2009; Andrade et al. 2012; MNHN 2012; Dietzen et al. 2014) have suggested lists of real focal bird species for European crop scenarios. However, few of these focus on the scenario of newly drilled treated cereal seeds, and none do so with specific reference to France.

In this paper, we present the results of extensive field observations of newly drilled cereal fields in France in autumn over 2 seasons, with knowledge gained in the first field season applied to refine and improve the efficiency of data collection in the second season. The data gathered have been used to identify bird focal species relevant for refined risk assessments for birds on French cereals during the autumn drilling period.

## MATERIALS AND METHODS

### Crop types and study sites

**Figure 1:**
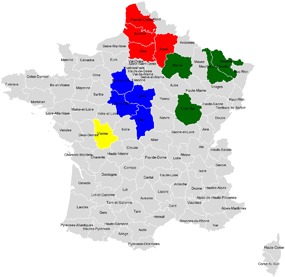
Distribution among French regions of field sites used in 2011 surveys (French departments containing field sites in North in red, in Center in blue, in East in green, and in West in yellow).

In 2011, sites were located in 13 French departments (French administrative areas), representing the main cereal production areas in northern, central, eastern, and western parts of France. Sixty‐five wheat and 21 barley fields, owned by 40 farmers, were selected, of which 44 were drilled with imidacloprid‐treated seed, that is, Gaucho® 350 or Ferial® (the ‘treatment’ fields), and the remaining 42 were drilled with seed to which other treatments (but not imidacloprid) had been applied (the ‘alternative treatment’ fields). As far as possible, fields were selected by pairs (with one treatment field selected close to one alternative treatment) to prevent the data set from being biased in favor of the most prevalent seed treatments used by farmers in France. Seeds were drilled at all sites by local farmers according to good agricultural practices. The 2011 autumn drilling season was sunny and dry throughout; some farmers postponed drilling owing to very dry soil conditions. Drilling of the study fields began on September 22 and continued until late October; the last bird observation session was on November 3. The distribution of sites among regions is shown in Figure 1 and Table 1. Sites were selected to be representative of French agriculture in general in terms of drilling machinery, rates, depths, and soil types.

**Table 1:** Distribution of survey sites across France during 2011 field season^a^

Region	French departments (code)	Wheat (‘treatment’ fields)	Wheat (‘alternative’ fields)	Barley (‘treatment’ fields)	Barley (‘alternative’ fields)	Total
North	Pas de Calais (62) Oise (60) Aisne (02) Somme (80)	15	10	3	3	31
Center	Eure‐et‐Loir (28) Cher (18) Loir‐et‐Cher (41) Loiret (45)	10	12	2	3	27
East	Marne (51) Meurthe‐et‐Moselle (54) Moselle (57) Côte d'Or (21)	4	6	6	4	20
West	Vienne (86)	4	4	0	0	8
	Total	33	32	11	10	86

^a^ “Treatment fields” refers to fields drilled with imidacloprid‐treated seeds; “alternative fields” refers to fields drilled with seeds to which other treatments have been applied.

With the knowledge from the 2011 work, sites for the second survey in 2012 were concentrated in the Centre and Picardie regions (Table 2) because during the drilling period of cereals, they had greater abundances of birds than elsewhere, so they represented higher potential risk to birds from treated seeds and allowed more efficient data collection. Twenty grouped fields were selected in each of these regions. Fields were all drilled with imidacloprid‐treated wheat seeds (because it was established from the 2011 data that seed treatment did not influence the bird community using the fields). The drilling period was from September 28 to October 23 in the Region Picardie and from September 29 to October 30 in the Region Centre.

**Table 2:** Distribution of survey sites across France during 2012 field seasons^a^

Region	French departments	Wheat (‘treatment’ fields)
Centre	Eure‐et‐Loir (28)	20
Picardie	Oise (60), Somme (80)	20
	Total	40

^a^ “Treatment fields” refers to fields drilled with imidacloprid‐treated seeds.

### Bird surveys

In 2011 each site was observed by ornithologists twice: once before and once within the 5 days after the drilling, to record those bird species present on the field and therefore potentially exposed to any cereal seeds remaining on the soil surface after drilling. A point‐count method was used: for 15 minutes the whole visible surface of the field was searched with a telescope or binoculars. To minimize disturbance, birds were observed from a vehicle used as a hide. All birds seen were recorded with the following information: species and number of individuals. Only data on birds observed in the cereal fields were used for real bird focal species determination. The same method was used in 2012 except that each site was observed once before and 4 (or 5) times after drilling on days +1, +2, +3, (+4), and +5 (or maximum +6). If observations were prevented by unfavorable conditions such as poor weather or hunting activity, observations were postponed for 1 day. If birds were not apparently disturbed by the arrival of the observers, observations began immediately; if disturbance was evident, observers waited a few minutes before commencing observations. Each observation on each field consisted of two 15‐minute periods. For small fields, these consecutive periods of observation were carried out from the same location, whereas for larger ones, the observers moved for the second period of observation to allow all parts of the field to be observed.

### Seed counts

On day 0 (i.e., drilling day) or maximum +4 (i.e., day 4 after the drilling date), on each field, 60 quadrats (1 m × 1 m) on 6 transect lines with a length of 50 m each were randomly chosen (2 in midfield area, 4 in headland areas). Every 5 m along each line a frame of 1 m^2^ was placed, and the number of visible seeds inside this frame was counted to give an estimate of exposure of birds to remaining seeds after drilling. Seeds counts were systematically performed on the cereal fields observed by ornithologists in 2011 and 2012.

### Data compilation and statistics

Correlation analyses using the generalized linear modeling with quasi‐Poisson distribution and negative binomial distribution were performed with seed count data and with bird monitoring data to assess the attractiveness of cereal seeds to birds for the years 2011 and 2012, respectively. For 2012, the average bird counts of the several surveys after drilling was correlated with the average headland and midfield seed count data.

In‐field data obtained in 2011 were examined for differences between barley and wheat, between imidacloprid‐treated fields and those with other treatments, and between fields before and after drilling, in terms of bird abundance, species richness, and diversity. All 2012 field data were collected from wheat treatment fields before and more than once after drilling (i.e., 4 dates of bird observations, and 5 dates exceptionally); the variable examined was the effect of time before and within the 5 days after drilling on bird abundance, species richness, and diversity. The measures used for statistical analyses were as follows:

- Abundance: the total number of individuals of a given species present on fields in a particular group;
- Species richness: the number of different species present on fields in a particular group;
- Shannon–Weaver diversity index *H* where

(1) $$H=\Sigma_{i=1}^{S}p_{i}\;\log_{e}p_{i}$$

- Simpson diversity index *D*, where

(2) $$D_{1}=1-\Sigma_{i=1}^{S}\;{p_{i}^{2}}_{}$$

in which *p_i_* is the proportion of species *i* and *S* is the number of species.

Linear mixed‐effect models were fitted to the whole data set with log transformation applied to total bird abundance and species richness.

For 2011 data, models with and without “crop” as a covariate were compared with a likelihood ratio test to determine whether there were differences between wheat and barley fields in terms of abundance, species richness, Shannon–Weaver Diversity Index, and Simpson's Diversity Index of the birds observed there.

Significant differences between barley and wheat fields were found with the 2011 data set (see *Results*); therefore, further assessments were made separately for each crop type (see *Results*). Because there were only 9 barley fields with nonzero observations, analysis of the effect of drilling (before and after drilling compared) and of seed treatment types was carried out only with the data from wheat fields, on which many more birds were seen (see *Results*). Mixed‐effect models were fitted to this data, followed by likelihood ratio tests to determine whether there was a significant effect of drilling (before or after) or treatment (treatment fields or alternative treatment fields) on the total abundance, species richness, and diversity of the bird species observed.

The same analysis of the effect of drilling (before or after) on the total abundance, species richness, and diversity of bird species observed was also done with the 2012 data set. Bird abundance and species‐richness data were also plotted on a log10 scale.

The same analysis procedures were applied to subsets of 2011 and 2012 data with either granivorous, omnivorous, carnivorous, or insectivorous species to test whether there were effects specific to these diet guilds. Species were assigned to guilds that are not mutually exclusive. For these statistical analyses, it was considered relevant to categorize a bird species as granivorous as soon as seeds, including cereal seeds, form part of its diet during the monitoring period (e.g., for the statistical analysis of the total abundance, species richness, and diversity of the bird species observed only, carrion crow, gray partridge, and wood pigeon were categorized as granivores (because of their preference for cereal seeds during the winter period) instead of omnivores although they have a mixed diet).

### Focal species selection criteria

Focal species determination was done in a consistent way by taking account of frequency of occurrence (FO), dominance, diet guild, and body weight [according to Dunning (2008)]. FO in a crop reflects the attractiveness of the crop for the species. The FO can be expressed in 2 ways: EFSA (2009) define FO_field_ as the number of fields in which a defined species was recorded as a percentage of the total number of fields, regardless of the number of individuals observed. That is, if a species is observed in 23 of 40 different fields (census sites), the frequency of occurrence per field (FO_field_) is 57.5%. This approach serves as a measure for the spatial prevalence, or the proportion of fields on which a species is present. If more than a single census is done on each field, FO_survey_ can provide information on the temporal evenness of occurrence of a species throughout the complete monitoring period. FO_survey_ is defined as the number of surveys in which a defined species was recorded as a percentage of the total number of surveys. For example, if 40 fields are visited 5 times during the monitoring period and a species is observed in 55 of 200 surveys (5 × 40), the FO_survey_ is 27.5%. Dominance is calculated as the number of individuals of a given species observed in all surveys on a field, as a percentage of the total number of individuals counted in all surveys on this field. These parameters were calculated for each combination of crop and species. As per EFSA's (2009) guidance, those species with FO values of 20% or more are considered to be of high priority, especially if they have high dominance, for the determination of candidate focal species.

Diet influences exposure through the differences in residues on different food types and the food ingestion rate, through the calorific value and assimilation efficiency on that food (Crocker et al. 2002). Therefore, all observed species were categorized into different diet guilds: carnivorous, insectivorous, granivorous, herbivorous, and omnivorous, as recommended by EFSA (2009). The allocation of a given species to a particular dietary guild is not exclusive. Body weight influences exposure predictions by allometric daily energetic requirements and thus food ingestion rate (Crocker et al. 2002); consequently, bird species with the lowest body weight among high‐ranking species within the guild are considered as the most appropriate focal species during the survey period.

The final selection process consisted of determining the most relevant focal species per diet guild, based on the geometric mean of FO_field_ values 20% or higher in 2011 and 2012 in order to consider all results from the surveys available from a given crop scenario in both years. Dietzen et al. (2014) apply these criteria successively (filtering process): select surveyed species with a mean FO_field_ higher than 20%, (from all the surveys available to them from a given crop scenario); then, select the species from each feeding group with the lowest body weight (exclude candidates with body weight more than 2 times the candidate with the lowest body weight); then, select the species with the highest FO (exclude candidates with an FO less than 0.5 of the candidate with the highest FO); and finally, select the species with the lowest body weight once again if necessary, to arrive at a single focal avian species for each crop scenario and feeding group.

## RESULTS

### Correlation of bird abundance with surface seed count

In 2011, in most of the bird surveys that were performed after the drilling, no birds were observed in field.

**Figure 2:**
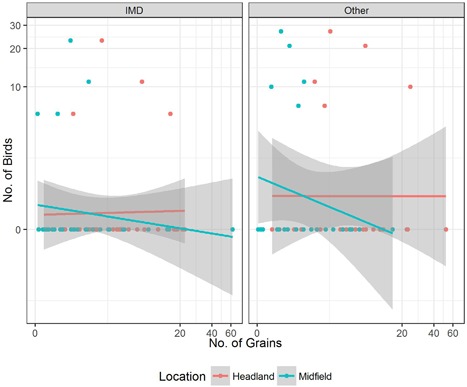
Surface seed counts versus bird counts, all fields monitored in 2011 (dots represent the number of birds observed in a survey; lines are regression curves indicating no significance at the statistical analysis).

**Figure 3:**
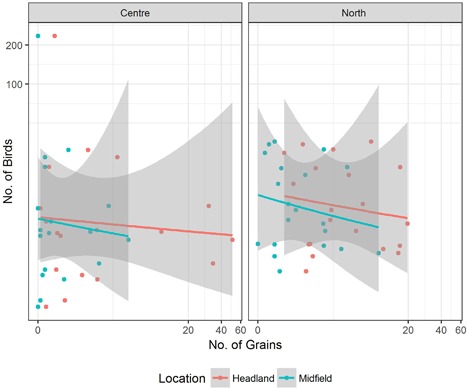
Surface seed counts versus bird counts, all fields monitored in 2012 in the 2 French regions located in the “Center” and “North” of France (Centre, Picardie) (dots represent the number of birds observed in a survey; lines are regression curves showing no significance at the statistical analysis).

The statistical evaluation did not show any significant relationship between the numbers of seeds on the soil surface of study fields and the number of birds observed in the headland and in the midfield for both years (Figure 2 and Figure 3).

### Barley versus wheat

The statistical analysis showed a significant difference between barley and wheat fields in terms of abundance, richness, and the 2 diversity indices if all bird data were used and if granivorous bird data only were used (*P* < 0.05; in Supplemental Data, see Table S1).

In 2011, of the 21 barley fields that were monitored, birds were observed on 10 barley fields only. Birds of only 4 species were observed, carrion crows being recorded on 6 fields out of the 9 (in Supplemental Data, see Table S2). Irrespective of the field and observation time (before or after drilling or treated with imidacloprid or not), few carrion crows were observed on the barley fields. On only 1 field was 1 carrion crow observed both before and after drilling.

One flock of pied wagtails was observed before drilling of the surveyed field (field 5). One flock of goldfinches was recorded on a different freshly drilled barley field (field 3). Two yellow‐legged gulls were also observed on 1 field (field 6).

Given the low number of barley fields that attracted birds, no further statistical analysis was directed at barley fields.

### Assessment of the “treatment” effect and the “drilling” effect with the 2011 data set

The bird monitoring data were examined for significant differences in abundance and diversity of birds between wheat treatment and alternative treatment fields and before and after drilling on each field category.

**Figure 4:**
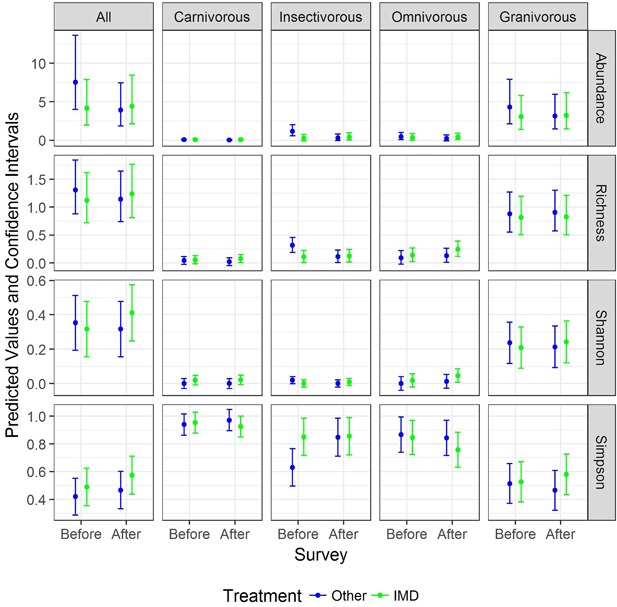
Predicted values and prediction confidence intervals of the abundance, richness, Shannon–Weaver index and Simpson index (each horizontal row) for the bird community containing carnivores, insectivores, omnivores and granivores (each vertical column) in wheat fields drilled with imidacloprid‐treated seed (‘IMD’) and those drilled with seed with other treatments (‘Other’) before and after drilling.

Statistical differences (*P* < 0.05) in abundance and species richness of birds were only observed for insectivorous birds when assessing the treatment effect (in Supplemental Data, see Table S3) and the drilling effect (in Supplemental Data, see Table S4). These statistical results can be explained by significantly higher numbers of individual birds and more insectivorous species in alternative treatment fields before they were drilled than in treatment fields or than in fields after they were drilled (as shown in Figure 4).

For all other diet guilds, abundance, species richness, and diversity indices were not significantly influenced by the treatment effect or the drilling effect.

### Assessment of the time effect before and after drilling with the 2012 data set

**Figure 5:**
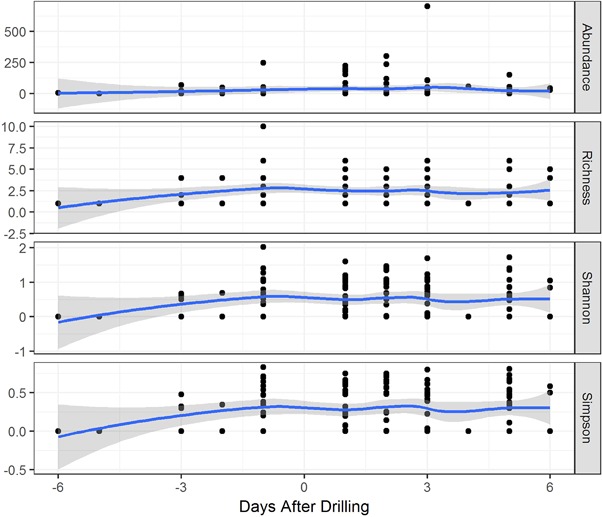
Estimated mean value and confidence bands (95% confidence level) of 4 metrics of bird abundance and diversity for all species observed on French wheat fields in autumn 2012 in relation to drilling date.

The modeled mean values of 4 measures of bird abundance and diversity were plotted against the time before and after drilling (Figure 5). A significant difference was observed between the surveys conducted before and after the drilling in the bird abundance, species richness, and Simpson's index for insectivorous birds only in 2012 (in Supplemental Data, see Table S5).

### Identification of candidate focal species

#### Barley

Four bird species were observed in 9 of the 21 barley fields. The carrion crow was the bird species most frequently observed on barley fields (Table 3). The dominance of goldfinches and pied wagtails in barley fields can be explained by the tendency of these species to form large flocks outside the breeding season.

**Table 3:** Frequency of occurrence per field, frequency of occurrence in surveys, and dominance for birds observed on French barley fields, autumn 2011

Species	FO_field_ (%)	FO_survey_ (%)	Dominance (%)	Diet guild
Carron crow *Corvus corone*	**28.6**	18.4	27.7	O
Goldfinch *Carduelis carduelis*	4.8	2.6	42.6	G
Yellow‐legged gull *Larus michaelis*	4.8	2.6	4.3	O
Pied wagtail *Motacilla alba*	4.8	2.6	25.5	I

^a^Values of FO_field_ ≥ 20% are in bold letters.

#### Wheat

Table 4 shows the values of FO_field,_ FO_survey_, and dominance calculated from the data collected from 65 fields in 2011 and 40 fields in 2012, and, considering the data collected in both years, the geometric mean of FO_field_ values.

**Table 4:** Frequency of occurrence per field, frequency of occurrence in surveys, and dominance for birds observed on French cereal fields in autumn 2011 and 2012 during drilling^a^

	2011			2012					
Species	FO_field_	FO_survey_	Dominance	FO_field_	FO_survey_	Dominance	Geomean FO_field_ (2011 and 2012)	Diet guild	Body weight (g)^b^
Carrion crow *Corvus corone*	**47.7**	**25.6**	5.1	**85**	**37.5**	13.2	**63.7**	O	570
Wood pigeon *Columba palumbus*	**27.7**	16.3	8.2	**42.5**	13.5	4.1	**34.3**	H/G	490
Common starling *Sturnus vulgaris*	**23.1**	12.4	**19.8**	**37.5**	11.5	**38.7**	**29.4**	O	79.9 (f)
Pied wagtail *Motacilla alba*	16.9	8.5	4.6	**35**	12.5	9.2	**24.3**	I	21
Gray partridge *Perdix perdix*	**30.8**	16.3	8.4	**30**	11	4.9	**30.4**	H/G	381 (f)
Skylark *Alauda arvensis*	**24.6**	13.2	3.2	**22.5**	6.5	7.1	**23.5**	O	33 (f)
Kestrel *Falco tinnunculus*	7.7	3.9	0.2	**22.5**	5.5	0.4	13.2	C/I	186 (m)
Pheasant *Phasianus colchicus*	9.2	4.7	0.8	**20**	6	0.6	13.6	H	953 (f)
Linnet *Carduelis cannabina*	10.8	6.2	9.5	**20**	5	3	14.7	G	15
Rook *Corvus frugilegus*	16.9	10.1	2.4	**20**	5	0.6	18.4	O	488
Chaffinch *Fringilla coelebs*	9.2	4.7	3.1	17.5	6	1.2	12.7	G/I	21 (f)
Feral pigeon *Columba livia*	1.5	0.8	0.2	17.5	4.5	4.1	5.1	G/H	340 (f)
Blackbird *Turdus merula*	6.2	3.1	0.4	15	6.0	1	9.6	O	113
Song thrush *Turdus philomelos*	–	–	–	15	4	0.3	–	O	66.6 (f)
Meadow pipit *Anthus pratensis*	3.1	1.6	0.1	15	3	0.4	6.8	I	18
Buzzard *Buteo buteo*	3.1	1.6	0.1	10	3.5	0.2	5.6	C	781 (m)
Lapwing *Vanellus vanellus*	7.7	4.7	8.9	10	2	6.9	8.8	I	211 (m)
Wheatear *Oenanthe oenanthe*	9.2	4.7	0.4	10	2	0.1	9.6	I	31
Magpie *Pica pica*	6.2	3.9	0.5	7.5	3	0.8	6.8	O	166 (f)
Robin *Erithacus rubecula*	3.1	1.6	0.1	7.5	2.5	0.2	4.8	O	18
Lesser black‐backed gull *Larus fuscus*	3.1	1.6	0.9	7.5	2	0.5	4.8	O	662 (f)
Corn bunting *Emberiza calandra*	–	–	–	7.5	1.5	0.2	–	G	46
Black‐headed gull *Chroicocephalus ridibundus*	1.5	1.6	5.2	7.5	1.5	0.1	3.4	O	284
Black redstart *Phoenicurus ochruros*	1.5	1.6	0.1	5	2	0.2	2.7	I	17
Jay *Garrulus glandarius*	6.2	3.1	0.2	5	1.5	0.1	5.6	O	161
Yellow‐legged gull *Larus cachinnans michaelis*	6.2	2.3	12.6	5	1	0.2	5.6	O	1033 (f)
Mistle thrush *Turdus viscivorus*	3.1	1.6	0.1	5	1	0.1	3.9	O	115
Yellowhammer *Emberiza citrinella*	1.5	0.8	0.1	5.0	1	0	2.7	G	27
Brambling *Fringilla montifringilla*	–	–	–	5	1	0	–	G/I	24
Red‐legged partridge *Alectoris rufa*	3.1	1.6	0.3	2.5	1	0.1	2.8	H/G	391 (f)
Stock dove *Columba oenas*	–	–	–	2.5	1	0.1	–	G/H	280 (f)
Great tit *Parus major*	–	–	–	2.5	1	0.1	–	G	19
Goldfinch *Carduelis carduelis*	1.5	0.8	0.03	2.5	0.5	3	1.9	G	16
House sparrow *Passer domesticus*	1.5	0.8	0.7	2.5	0.5	0.4	1.9	G	27
Jackdaw *Corvus monedula*	1.5	0.8	0.1	2.5	0.5	0.2	1.9	G/O	246
Herring gull *Larus argentatus*	4.6	3.1	1.9	2.5	0.5	0.1	3.4	O/C	1044 (f)
Whinchat *Saxicola rubetra*	–	–	–	2.5	0.5	0.1	–	I	16.6
Collared dove *Streptopelia decaocto*	1.5	0.8	0.8	2.5	0.5	0.1	1.9	G/H	146 (f)
Greenfinch *Carduelis chloris*	1.5	0.8	0.03	2.5	0.5	0	1.9	G/I	28
Peregrine falcon *Falco peregrinus*	–	–	–	2.5	0.5	0	–	C/I	611 (m)
Great black‐backed gull *Larus marinus*	–	–	–	2.5	0.5	0	–	O	1488 (f)
Dunnock *Prunella modularis modularis*	–	–	–	2.5	0.5	0	–	G	19.7
Fieldfare *Turdus pilaris*	1.5	0.8	0.1	2.5	0.5	0	1.9	O	104 (f)

^a^ Values of 20 or more are in bold type. The species in the upper part of the table are those selected as candidate focal species for autumn‐drilled cereal seed treatments in France. The species appear in order of descending FO_field_, then ascending body weight. The table should be used by prioritizing members of a given feeding guild closest to the top of the table, bird species with the geometric mean of the FO_field_ ≥ 20% being shaded.

^b^ Where m or f is specified, the mass of the smaller sex is given (Dunning 1993).

The selected focal species for autumn‐drilled cereal seed treatments are those in the upper part of Table 4 underlined in gray. The species appear in order of descending FO_field_, then ascending body weight. The table should be used by prioritizing members of a feeding guild closest to the top of the table (“candidate focal species”). Table 5 shows results of the analysis performed according to the filtering process detailed by Dietzen et al. (2014). This results in the selection of skylark (omnivores), gray partridge (herbivores and granivores), wood pigeon (herbivores and granivores), and pied wagtail (insectivores) as the relevant focal species for assessing the risk to birds in winter cereals in France, from September to November.

**Table 5:** Relevant focal species determination in winter cereals considering geomean FO_field_ value ≥ 20% (focal species candidate) from the 2 consecutive years (2011 and 2012) in winter cereals and following the filtering process detailed in Dietzen et al. (2014)

	Filter 1	Filter 2	Filter 3	Filter 4
Diet guild	geomean FO_field_ ≥ 20%	lowest bw	highest FO	lowest bw
Granivorous	Carrion crow	Skylark	Skylark	Skylark
	Common starling			
	Skylark			
Herbivorous	Wood pigeon	Wood pigeon	Wood pigeon	Wood pigeon
	Gray partridge	Gray partridge	Gray partridge	Gray partridge
Granivorous	Wood pigeon	Wood pigeon	Wood pigeon	Wood pigeon
	Gray partridge	Gray partridge	Gray partridge	Gray partridge
Insectivorous	Pied wagtail	Pied wagtail	Pied wagtail	Pied wagtail

bw = body weight; FO = frequency of occurrence.

## DISCUSSION

### Wheat versus barley and bird abundance

Significantly fewer birds, and fewer species, were seen in barley fields than in wheat fields in 2011, and for this reason the survey work in 2012 concentrated on wheat fields. Practices in drilling wheat and barley are very similar (although barley is usually drilled slightly earlier), and the surveys indicate that the species selected as (candidate) focal species from observations on wheat fields will adequately represent those likely to occur on barley fields. Those species seen on barley fields in our surveys were all also seen on wheat fields, the carrion crow being the most frequently observed in barley and wheat fields in our study and by Crocker and Irving (1999). This conclusion is supported by the surveys of CSL (2009), which concentrated on the period immediately after drilling of wheat and barley seeds and produced almost identical “top 10” lists of species for the 2 crops.

### Seed treatments and bird abundance

There were no statistically significant differences in bird abundance between treatment and alternative treatment fields, except in the case of insectivorous birds, before fields were drilled. At this stage, there cannot be any influence of the seed treatment and hence this significant difference is not attributable to the seed treatment. Although it might be expected that birds would visit a newly drilled field to consume the drilled seeds, the abundance of birds on the fields tended to be lower after the fields had been drilled. No relationship between the number of seeds on the soil surface and the number of birds observed in the headland and in the midfield areas was observed, showing the limited attractiveness of remaining seeds to birds. Typically, drilling is the last in a succession of cultivation processes in which seedbed preparation is an important step; these processes (prior to drilling) may attract birds because they disturb and bring to the soil surface invertebrates and weed seeds that were previously unavailable. If crop seeds are then drilled accurately into a well‐prepared seedbed, little food remains available after drilling. For many birds looking for food during this period of the year, the better availability of food items on other fields may be an explanation of the limited attractiveness of conventional winter cereal fields (Wilson et al. 1996; Eraud et al. 2015).

### Bird survey methods

Different bird survey methods exist that may influence results of bird monitoring activities and focal species determination. EFSA (2009) suggests 2 methods for surveying birds in cropped fields: transect counts, in which an observer moves along a line through a crop and field, recording birds by sight or sound within a defined distance of the transect line, and point‐count methods, in which the observer remains stationary at a fixed point. EFSA indicates that either method is valid but that the point‐count method may be more appropriate for freshly drilled fields or bare soil, where vegetation does not obscure birds at a distance from the observer. Both methods are subject to the risk that observers may overlook small birds at a great distance from either the transect line or the observation point, particularly where there is some ground cover or the soil surface is heterogeneous.

Muséum National d'Histoire Naturelle (MNHN) (2012) used both transect survey data from regulatory study surveys conducted as per EFSA (2009) guidelines, and point‐count survey data from the French Breeding Bird Survey (FBBS) (Suivi Temporel des Oiseaux Communs, STOC—trends in common birds) to identify focal species in France, depending on the crop and the data available on pesticide applications. These methods were considered as complementary. Andrade et al. (2012) used existing point‐count data from the FBBS (Jiguet et al. 2012), focusing on cereals, to adapt an index, IndVal (Dufrêne and Legendre 1997) of habitat specificity for farmland species by combining it with FO data. Jiguet et al. (2012) decided to minimize detection bias by visiting each counting point twice. A transect‐count method would have been less vulnerable to this detection bias, and 2 surveys per site is a small sample. Additionally, at each point‐count, in the monitoring program of the FBBS, the surveyed area was not exclusively a cropped area as in the 86 cereal fields we surveyed for this paper or as described for focal species determination according to EFSA (2009) guidelines.

Wilson et al. (1996) and Crocker and Irving (1999) addressed the problem of the transect‐count method by using a “complete count” or “total flush” method: the observer used binoculars and walked through the field to observe every part of it and to disturb, and thus see and count, every bird present. The observer kept walking until satisfied that every bird had been accounted for. This method allows better comparisons of the bird abundance and composition in different crops because clearly a point‐count method may underestimate the number of birds in a tall, dense crop and more accurately estimate those present on bare, dark soil. Crocker and Irving (1999) compared bird numbers on different crops by using this method. Wilson et al. (1996) also described the necessity of surveying several different crops in a way that made the figures unbiased by the nature of the crop. However, this approach was not necessary in our study, in which only newly drilled fields were surveyed.

Bird censuses were performed by Lopez‐Antia et al. (2016) shortly after finishing sown seed sampling of cereals (from October to December). Each census covered a large area of approximately 4 hectares per field and was conducted for 30 minutes between 8:30 am and 10:30 am. However, only bird species feeding in the freshly drilled field were counted. It differs from our approach in which all birds observed in the field during the survey period were recorded.

The point‐count method has the advantage of being relatively rapid and efficient. And, according to our knowledge, with our study, it was the first time that so many fields were surveyed within a short window of time centered on drilling and in different French regions. Naturally, when conditions are favorable in an area, many farmers will be drilling simultaneously. A survey method that allows a surveyor or survey team to carry out several surveys per day will thus be more efficient in these circumstances and allow a greater number of fields to be included.

The main weakness of field surveys described in the literature is that they have been carried out over a long period, encompassing many stages of crop development. However, cereals are drilled into bare soil or stubble, and this practice presents a very different habitat for birds to that of an established crop. Comparison of Crocker and Irving (1999) and CSL (2009) shows that different species are found on newly drilled fields compared to those observed in crops over a longer period. Therefore, only surveys focused on newly drilled fields are appropriate to select focal species for use in assessment of risk from seed treatments.

### Metrics used to identify candidate focal species

EFSA suggests prioritizing species with FO values of 20% or higher. Dietzen et al. (2014) consider the effects of varying this threshold and conclude that 20% guarantees that a focal species that occurs only in one‐fifth of all fields is identified in 90% of cases and that a higher threshold would increase the probability that a relevant focal species is not detected or that a lower threshold would inflate the list of focal species beyond its usefulness as a risk‐based filter.

MNHN (2012) used datasets from the FBBS (Jiguet et al. 2012) and from industry surveys conducted according to EFSA (2009) guidelines. A filter of FO of 10% or more was applied to obtain a list of candidate focal species from each data set. The 2 lists were then compared, and the species common to both selected as candidate focal species. When there was only one data set available for a crop, all the species with FO of 10% or more were included in the list of candidate focal species. MNHN (2012) argued that in many cases, 20% did not identify a sufficient number of species when the 2 data sets were combined. A second level of filtering was then applied, considering the candidate species’ diet, feeding guild, and body mass, selecting in each case the smallest representative of each feeding guild or ecological group.

Crocker and Irving (1999) and CSL (2009) calculate and present abundance (the number of individual birds of each species per 10 ha observed, averaged for each crop) and prevalence (the proportion of surveys on which a species was seen, regardless of the number of individuals present on each occasion). Surveys were carried out over the course of a year on more than 200 fields in 2 areas of English intensive arable agriculture. “Prevalence” is equivalent to FO_field_. They rank species for each crop by abundance and by prevalence, take account of both measures by adding the abundance and prevalence rank for each species, and then rerank by the sum of abundance and prevalence. They present lists by crop and season and for large and small birds separately.

Crocker and Irving's (1999) subset of data for cereals in autumn (split into wheat and barley) is comparable to our own, although they did not concentrate on the immediate drilling period. CSL (2009) did focus surveys in this period, and their data for cereals, generated in the UK, are directly comparable to our surveys in France (Table 6).

**Table 6:** Candidate focal species identified in this study and in other comparable studies

	Identified as candidate focal species for bare soil or new drill in				
Species	This study	Central Science Laboratory (2009)	Dietzen et al. (2014)	Dietzen et al. (2014)	Lopez‐Antia et al. (2016)
	FR	UK	Central zone (DE, PL, FR)	Southern zone (FR, IT, ES)	ES
Carron crow *Corvus corone*	✓	B, W		✓	
Wood pigeon *Columba palumbus*	✓	B. W			
Gray partridge *Perdix perdix*	✓				
Skylark *Alauda arvensis*	✓	B, W	✓		✓
Common starling *Sturnus vulgaris*	✓	B, W			
Pied wagtail *Motacilla alba*	✓				
Rook *Corvus frugilegus*	✓	B, W			
Linnet *Carduelis cannabina*	✓				
Kestrel *Falco tinnunculus*	✓				
Pheasant *Phasianus colchicus*	✓	W			
Black‐headed gull *Chroicocephalus ridibundus*		B, W			
Lapwing *Vanellus vanellus*		B, W			
Yellow wagtail *Motacilla flava*			✓	✓	
Corn bunting *Miliaria calandra*					✓
Crested lark *Galerida cristata* L.					✓
Spanish sparrow *Passer hispaniolensis* T.					✓

^a^B and W denote species identified for barley and wheat, respectively (when these crops are considered separately; in other studies, they are grouped as “cereals”). Species identified from non‐French studies as focal species in only 1 column or with FO <20% are omitted from this table for brevity and clarity.

B = barley; DE = Germany; ES = Spain; FR = France; IT = Italy; O = omnivorous; PL = Poland; W = wheat.

Lopez‐Antia et al. (2016) performed 89 bird censuses in 23 cereal fields around the Tablas de Daimiel National Park in the region of La Mancha in Spain's southern central plateau; in their final database used for statistical analysis, they included only species that were visually confirmed to be picking up seeds and shoots up during the bird observations. They decided to focus their analysis on feeding birds in cereal fields, which may limit the number of bird species of concern compared to our approach.

### Identification of final focal species

EFSA (2009) points out that “the survey data may be analyzed in a variety of ways, however in trying to determine ‘focal species’ the following information is considered to be most relevant: FO_field_ and FO_survey_. Those species with a FO higher than 20% might be considered to be of high priority especially if they have high dominance. However, it is necessary to consider issues such as feeding strata, food intake rate, body weight of potential focal species and diet to ensure that species with the highest potential exposure are considered.” In this way, species that may also use the crop but that have a lower potential exposure should be protected by a risk assessment for the focal species.

In our study, we applied the Dietzen et al. (2014) filtering process to determine relevant focal species. However, differences in body weight of the wood pigeon and the gray partridge are low in the final step of the filtering process and so do not justify excluding either species as would otherwise have been done in the final step (refer to the “Focal species selection criteria”).

Focal species were identified for the southern and central zones in winter cereals (including France) by Dietzen et al. (2014). However, none of the studies are qualified for the immediate postdrilling period (bare soil).

Crocker and Irving (1999) predated and influenced the EFSA (2009) guidance. Although they have not used the term FO, their calculation of frequency and abundance and combined ranking of them is in line with EFSA's guidance, and their tables of large and small species with high ranks in each crop observed allows final candidate species to be identified. CSL (2009) used the same methods and calculations applied specifically to newly drilled crops to generate similar tables. CSL's candidate focal species for newly drilled cereals are the most comparable to our own, as reported in Table 5, probably because their surveys, like ours, focused on the immediate postdrilling period. Despite having been conducted in different countries and the use of slightly different approaches to identifying candidate focal species, the lists produced are very similar, suggesting that focusing on the immediate postdrilling period is important in determining focal species relevant to winter cereals.

Lopez‐Antia et al. (2016) recently reported frequent observations of skylarks on cereal fields in autumn in Spain, which is consistent with our results obtained on newly drilled cereal fields in autumn in France over 2 years (see Table 6). Our data confirmed the relevance of skylarks as a focal species in freshly drilled winter cereal fields in France (Table 4), with higher FO values in 2011 than in 2012. Indeed, France is one of the key wintering grounds for migrating skylarks originating from many parts of Europe (Hémery et al. 1992; Spaepen 1995; Henry et al. 2014); wintering sometimes habitually occurs with large flocks in winter wheat fields. Eraud et al. (2015) studied the seed fraction of the diet of Eurasian skylarks wintering in western France (from early December to late January on wheat fields, in particular). The seed content of each gizzard was inspected with a binocular microscope, and seeds were counted and identified on the basis of their size, shape, and color and with use of the reference collection held at the French National Institute for Agricultural Research (INRA). In contrast to Green (1978), Donald and Vickery (2000), and Donald et al. (2001), Eraud et al. (2015) did not find a single wheat seed in any skylarks’ gizzards in any region or period of the study. Whatever the region or period, weeds, not grain, were the predominant seeds. The explanation given was that cereal grain may have completely vanished from early December onwards and that this resource does not contribute at all to the diet of wintering skylarks (and other farmland birds) in modern farmlands. The results of Geiger et al. (2014) suggest that skylarks have a strong preference for cereal grains because of their high energy content. Therefore, skylarks probably take cereal grains if available and switch to other, less profitable, food types when the grain supply is depleted. MNHN (2012) reported skylarks to be present between August and November with a variable diet that is mainly herbivorous when grains are depleted (Green 1978; Robinson 2004).

Despite high FO_field_ and FO_survey_ values, application of criteria by Dietzen et al. (2014) exclude as focal species larger omnivorous birds such as crows, rooks, and starlings frequently observed in flocks in winter, which look for all foraging opportunities provided by the farming cycle but have no preference for winter cereal fields (Whitehead et al. 1995; Mason and MacDonald 2004). These species are frequently considered agricultural pests by farmers, leading to use of bird control methods to prevent crop damage.

Additionally, our results show consistency in exposure of game birds such as wood pigeons and gray partridges in freshly drilled winter wheat fields with high FO_field_ and FO_survey_ values (Tables, 4, and 6 3). Both species should be listed as focal species because of their considerable plasticity in feeding habits: they are able to change the main food item consumed over a short period of time, as reported in the French Cahier Agriculture Oiseaux (MNHN 2012, page 92). Moorcroft et al. (2002) showed that in autumn, gray partridges rarely fed on stubble fields where cereal grain density was below 50 seeds per m^2^, as is the case when good drilling practices are followed by farmers. Cereal seed consumption in freshly drilled fields by gray partridges may be influenced by regular releases of captive‐bred gray partridges in France, which may have different feeding behavior than wild gray partridges (i.e., after breeding, gray partridges are looking for game bird feeders within the fields).The wood pigeon was the second most frequently observed species in fields during our 2011 and 2012 bird surveys, with a higher abundance of wood pigeon than gray partridge. Their feeding habits (forming large flocks when a cereal field is used as a source of food), their capacity to collect seeds quickly, and their known large daily foraging range in the agricultural landscape, which generally contains stubble fields where cereals are drilled in autumn, might explain these results (Haynes et al. 2003).

Among the small granivorous species, the linnet occurs in the list of potential focal species in winter wheat fields in 2012, with a FO_field_ of 20%. However, this species was excluded from the final list owing to the FO_field_ value obtained in 2011 (FO_field_ 10.8%), which leads to a calculated geometric mean FO_field_ value lower than 20%. FO_survey_ was low in both years (Table 4). The results from both years were consistent because linnets occurred only in wheat fields in the same 3 French departments (60, 80, 28). We prioritized the results of 2011 owing to the higher number of monitored fields (65 versus 40) and the larger areas covered (11 departments in 2011 versus 3 departments). Moreover, linnets were only observed in those few fields in which the previous crop was oilseed rape and that had been recently plowed in preparation for winter cereal drilling. As linnets are known to favor such small seeds (CSL 2009), we referred these linnet observations as resulting from the presence of oilseed rape seeds from the previous crop on the drilled bare soil. We could not find any indications that drilled cereal fields were of any attractiveness to small granivorous birds and conclude that these birds should not be considered as obligatory species for assessing the risk for treated cereal seeds. However, linnets should not be excluded from a risk assessment for spray applications (pre‐ or early postemergence), although fields containing winter cereals on conventionally managed farms are relatively “clean” of most arable weed seeds, making them unattractive to linnets (Wilson et al. 1996; Crocker and Irving 1999; Hancock and Wilson 2003).

Lopez‐Antia et al. (2016) focused part of their bird observations on cereals after drilling; they suggest that the corn bunting could be a good focal species to estimate the risk from the ingestion of cereal seeds treated with pesticides. However, as stated by Dietzen et al. (2014), even if the corn bunting is a focal species for cereal fields in Spain and Portugal, it is not of relevance in France where cereals are mainly grown in the northern part of the country.

In winter most insectivorous birds either migrate, for example, warblers (Sylviidae), or change their diet to include more vegetable matter, for example, tits (Paridae) and buntings (Emberizidae). The pied wagtail is a partial migrant, classified sometimes as a subspecies of the white wagtail, which was frequently observed in winter wheat and barley fields in 2011 and 2012, leading us to identify it as a relevant focal bird species. At this period of the year, winter survival is primarily a matter of obtaining enough food. Davies (1982) found that pied wagtails needed to find 1 small insect every 4 seconds throughout the whole of the winter day for survival. Plowed fields can provide a rich source of invertebrates exposed at the soil surface, which may attract insectivorous birds like pied wagtails (Wilson et al. 1996) and which may explain the significantly higher bird abundance and species richness of insectivorous birds observed before wheat fields were drilled in 2011 and 2012.

## CONCLUSION

EFSA (2009) gives generic focal species for consideration in lower‐tier risk assessments for plant protection products but does not propose real focal bird species for refinement steps, applicable at country level. According to our knowledge, this is the first time that a large and pluriannual database of in‐crop field observations of birds has been generated and used to determine the most relevant bird species to freshly drilled fields of cereals in France for risk assessment. Although fewer individual birds and species were observed on winter barley fields than on winter wheat fields, no distinction is needed for the risk assessment. Carrion crow, wood pigeon, gray partridge, skylark, common starling, and pied wagtail were the bird species most frequently observed during the drilling period on winter cereal fields according to 2011 and 2012 monitoring data. This list is consistent with focal species proposals on newly drilled cereal fields in the UK (CSL 2009), frequently used as a reference in the regulatory process. The list of focal species candidates was processed to identify the most relevant focal species according to Dietzen et al. (2014), resulting in the selection of the skylark (omnivores), the gray partridge (herbivores and granivores), the wood pigeon (herbivores and granivores), and the pied wagtail (insectivores) as the relevant focal species for assessing the risk to birds from pesticide‐treated winter cereal seeds in France (September through November). The use of these focal species in refined risk assessments when needed at national level should significantly increase confidence and assist in the regulatory decision‐making process for registration of pesticides.

## SUPPLEMENTAL DATA

**Table S1.** *P* values for crop type (wheat or barley) derived from comparing a mixed‐effect model with crop as a covariate and the same model without crop by use of a likelihood ratio test.

**Table S2.** Results of bird observations performed in 2011 on barley fields (I = insectivorous; O = omnivorous; TA = field not drilled with imidacloprid barley seeds; TG = field drilled with imidacloprid barley seeds).

**Table S3.** *P* values for “treatment effect” (fields drilled with imidacloprid‐treated seed or fields drilled with dressed seeds with compounds other than imidacloprid) derived from comparing a mixed‐effect model with treatment as a covariate and the same model without treatment, by use of a likelihood ratio test; 2011 survey data.

**Table S4.** *P* values for “drilling effect” (before and after drilling) derived from comparing a mixed‐effect model with survey time as a covariate and the same model without survey time by use of a likelihood ratio test; 2011 survey data.

**Table S5.** *P* values for “drilling effect” (before and after drilling) derived from comparing a mixed‐effect model with survey time as a continuous covariate and the same model without survey time by use of a likelihood ratio test (*P < 0.05); 2012 survey data.

## Supporting information

This article includes online‐only Supplemental Data.

Supporting Tables S1.

## Data Availability

Data, associated metadata, and calculation tools are available upon request from the corresponding author, Emmanuelle Bonneris, at emmanuelle.bonneris@bayer.com.
