# Supplementary material for: Selecting appropriate focal species for assessing the risk to birds from newly drilled pesticide‐treated winter cereal fields in France
Source: Integr Environ Assess Manag. 2019 Feb 11;15(3):422–36. doi: 10.1002/ieam.4112 (PMC6850368; doi:10.1002/ieam.4112)
Supplement: Supplementary file 1 — Supporting Tables S1. [file IEAM-15-422-s001.docx]

**Supplemental material: Tables giving detailed results on statistics and results of bird observations performed in 2011 and 2012 on barley and wheat fields**

Table S1. P-values for crop type (wheat or barley) derived from comparing a mixed effect model with crop as a covariate and the same model without crop, using a likelihood ratio test.

|  | All diet guilds | Granivores | Insectivores | Omnivores | Carnivores |
| --- | --- | --- | --- | --- | --- |
| Abundance | 0.0002* | 0.0005* | 0.345 | 0.491 | 0.671 |
| Species richness | 3.778e-05* | 0.0002* | 0.164 | 0.165 | 0.637 |
| Shannon diversity index | 0.001* | 0.008* | 0.867 | 0.703 | 0.845 |
| Simpson diversity index | 0.004* | 0.004* | 0.171 | 0.248 | 0.683 |

*statistical difference observed in barley and wheat fields with p<0.05

Table S2. Results of bird observations performed in 2011 on barley fields (TG: field drilled with imidacloprid barley seeds; TA: field not drilled with imidacloprid barley seeds; O: Omnivorous, I: Insectivorous).

| Field Code | Status | | Survey | Species | Diet Guild | Number of individuals |
| --- | --- | --- | --- | --- | --- | --- |
| Field 1 | | TG | 2 | Carrion crow | O | 4 |
| Field 2 | | TA | 2 | Carrion crow | O | 2 |
| Field 3 | | TA | 2 | Goldfinch | G | 20 |
| Field 4 | | TG | 2 | Carrion crow | O | 3 |
| Field 5 | | TA | 1 | Pied wagtail | I | 12 |
| Field 6 | | TA | 1 | Yellow-legged gull | O | 2 |
| Field 7 | | TG | 1 | Carrion crow | O | 1 |
| Field 7 | | TG | 2 | Carrion crow | O | 1 |
| Field 8 | | TA | 1 | Carrion crow | O | 1 |
| Field 9 | | TG | 2 | Carrion crow | O | 1 |

**Table S3.** P-values for ‘treatment effect’ (fields drilled with imidacloprid-treated seed or fields drilled with dressed seeds with other compounds than imidacloprid) derived from comparing a mixed effect model with treatment as a covariate and the same model without treatment, using a likelihood ratio test – 2011 survey data.

|  | All diet guilds | Granivores | Insectivores | Omnivores | Carnivores |
| --- | --- | --- | --- | --- | --- |
| Abundance | 0.351 | 0.745 | 0.040* | 0.694 | 0.532 |
| Species richness | 0.786 | 0.920 | 0.044* | 0.397 | 0.457 |
| Shannon diversity index | 0.658 | 0.887 | 0.363 | 0.409 | 0.359 |
| Simpson diversity index | 0.434 | 0.508 | 0.062 | 0.592 | 0.641 |

*statistical difference observed in fields drilled with imidacloprid-treated seed or those drilled with seeds dressed with other compounds than imidacloprid with p<0.05

Table S4. P-values for ‘drilling effect’ (before and after drilling) derived from comparing a mixed effect model with survey time as a covariate and the same model without survey time, using a likelihood ratio test – 2011 survey data.

|  | All diet guilds | Granivores | Insectivores | Omnivores | Carnivores |
| --- | --- | --- | --- | --- | --- |
| Abundance | 0.278 | 0.772 | 0.035* | 0.635 | 0.822 |
| Species richness | 0.800 | 0.993 | 0.036* | 0.339 | 0.806 |
| Shannon diversity index | 0.655 | 0.884 | 0.367 | 0.547 | 0.996 |
| Simpson diversity index | 0.581 | 0.757 | 0.058 | 0.499 | 0.713 |

*statistical difference observed in fields before and after drilling with p<0.05

Table S5. P-values for ‘drilling effect’ (before and after drilling) derived from comparing a mixed effect model with survey time as a continuous covariate and the same model without survey time, using a likelihood ratio test (p*<0.05) – 2012 survey data.

|  | All diet guilds | Granivores | Insectivores | Omnivores | Carnivores |
| --- | --- | --- | --- | --- | --- |
| Abundance | 0.919 | 0.668 | 0.026* | 0.922 | 0.300 |
| Species richness | 0.838 | 0.666 | 0.029* | 0.514 | 0.454 |
| Shannon diversity index | 0.600 | 0.625 | 0.171 | 0.226 | Not Applicable |
| Simpson diversity index | 0.394 | 0.672 | 0.050* | 0.933 | 0.454 |

*statistical difference observed in fields before and after drilling with p<0.05
